# Rapid improvement of systemic sclerosis-associated intestinal pseudo-obstruction with intravenous immunoglobulin administration

**DOI:** 10.1093/rheumatology/kead093

**Published:** 2023-02-24

**Authors:** Kazuki M Matsuda, Ayumi Yoshizaki, Ai Kuzumi, Satoshi Toyama, Kentaro Awaji, Tomomi Miyake, Shinichi Sato

**Affiliations:** Department of Dermatology, The University of Tokyo Graduate School of Medicine, Tokyo, Japan; Department of Dermatology, The University of Tokyo Graduate School of Medicine, Tokyo, Japan; Department of Dermatology, The University of Tokyo Graduate School of Medicine, Tokyo, Japan; Department of Dermatology, The University of Tokyo Graduate School of Medicine, Tokyo, Japan; Department of Dermatology, The University of Tokyo Graduate School of Medicine, Tokyo, Japan; Department of Dermatology, The University of Tokyo Graduate School of Medicine, Tokyo, Japan; Department of Dermatology, The University of Tokyo Graduate School of Medicine, Tokyo, Japan

**Keywords:** scleroderma and related disorders, gastrointestinal, systematic review, immunotherapy, soft tissue rheumatism

## Abstract

**Objectives:**

SSc is an autoimmune disease characterized by excessive fibrosis in multiple organs, including the gastrointestinal (GI) tract. GI symptoms of SSc such as intestinal pseudo-obstruction (IPO) are often refractory to conventional intervention and can result in longer in-hospital stay or even increased mortality. We aimed to summarize the insights to date regarding the efficacy of IVIG against GI symptoms of SSc to unveil what we should focus on in future studies.

**Methods:**

Herein we report the response of GI symptoms in three cases with SSc-myositis overlap who received IVIG administration. We also conducted a systematic literature review to summarize previous reports regarding the efficacy of IVIG upon the GI manifestations of SSc, according to the PRISMA 2020 guideline.

**Results:**

The case series demonstrated remarkable and rapid improvement of GI symptoms, including IPO, after IVIG administration. The literature review revealed that previous reports also support the efficacy and safety of IVIG against GI manifestations of SSc. However, they were all retrospective studies and lacking description of the short-term outcome after IVIG administration with objective and quantitative metrics.

**Conclusion:**

IVIG seems to be a promising therapeutic option for the management of GI symptoms in SSc, including IPO. Investigators should focus more on short-term outcomes to properly assess the therapeutic benefit of IVIG, ideally using reliable quantitative measures in a multicentre randomized placebo-controlled setting.

## Graphical abstract

**Figure:**
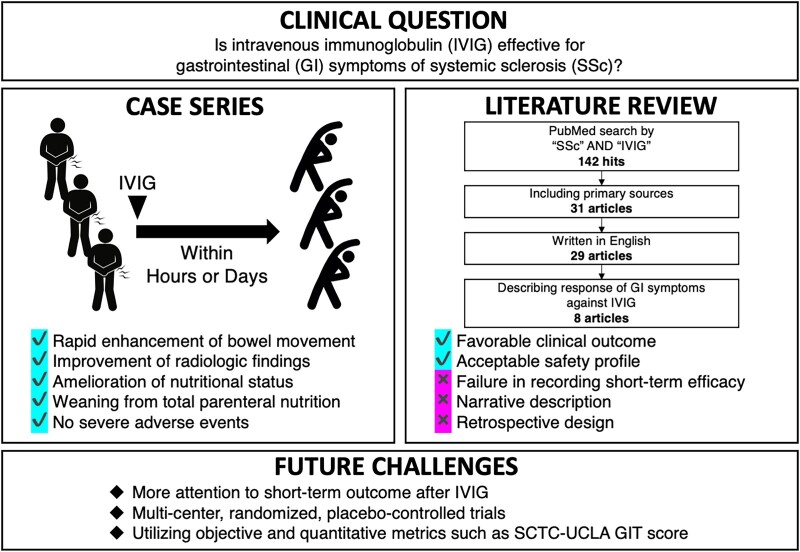

Rheumatology key messages

- Gastrointestinal symptoms of systemic sclerosis, including intestinal pseudo-obstruction, is often difficult to treat.
- Our case series and literature review supported efficacy of intravenous immunoglobulin in the short-term.
- Validation using quantitative measures in randomized placebo-controlled setting remains a challenge for future investigations.

## Introduction

SSc is an autoimmune disease characterized by excessive fibrosis in multiple organs, such as the skin, lung and gastrointestinal (GI) tract [1]. GI symptoms, including reflux, distention, diarrhoea and constipation are suffered by almost all SSc patients and critically impair their quality of life [2]. Hypomotility of the GI tract leads to dysphagia and intestinal pseudo-obstruction (IPO), which sometimes result in recurrent aspiration pneumonia, pneumatosis cystoides intestinalis (PCI), a requirement of total parenteral nutrition (TPN), longer in-hospital stay, or even increased mortality [3]. These conditions are often unresponsive to conventional interventions such as immunomodulators, prokinetics, probiotics, and oral antibiotics. Therefore, the management of GI manifestations of SSc, especially SSc-associated IPO (SSc-IPO), is crucial for their prognosis and remains a challenge for healthcare providers.

IVIG is one of the immunomodulatory agents being tried for SSc management, characterized by its low adverse event profile [4]. Although the pharmacodynamics has not yet been fully understood, previous studies have suggested the beneficial effect against multiple aspects of SSc manifestations, including skin sclerosis, lung fibrosis and myopathy. IVIG is particularly considered as a therapeutic option in SSc-myositis overlap syndrome, because a number of previous studies including multiple randomized, placebo-controlled trials have supported the benefit of IVIG against inflammatory myopathies [5].

We experienced three cases of SSc-myositis overlap in which remarkable and immediate improvement of SSc-IPO was observed after IVIG administration. Herein we describe those cases and the result of the systematic literature review to summarize previous reports. Our aim is to discuss the potential therapeutic effect of IVIG for GI manifestations of SSc, especially focusing on its rapid onset after administration.

Informed consent was received for publication of this manuscript.

## Case presentation

### Case 1

**Figure 1.**
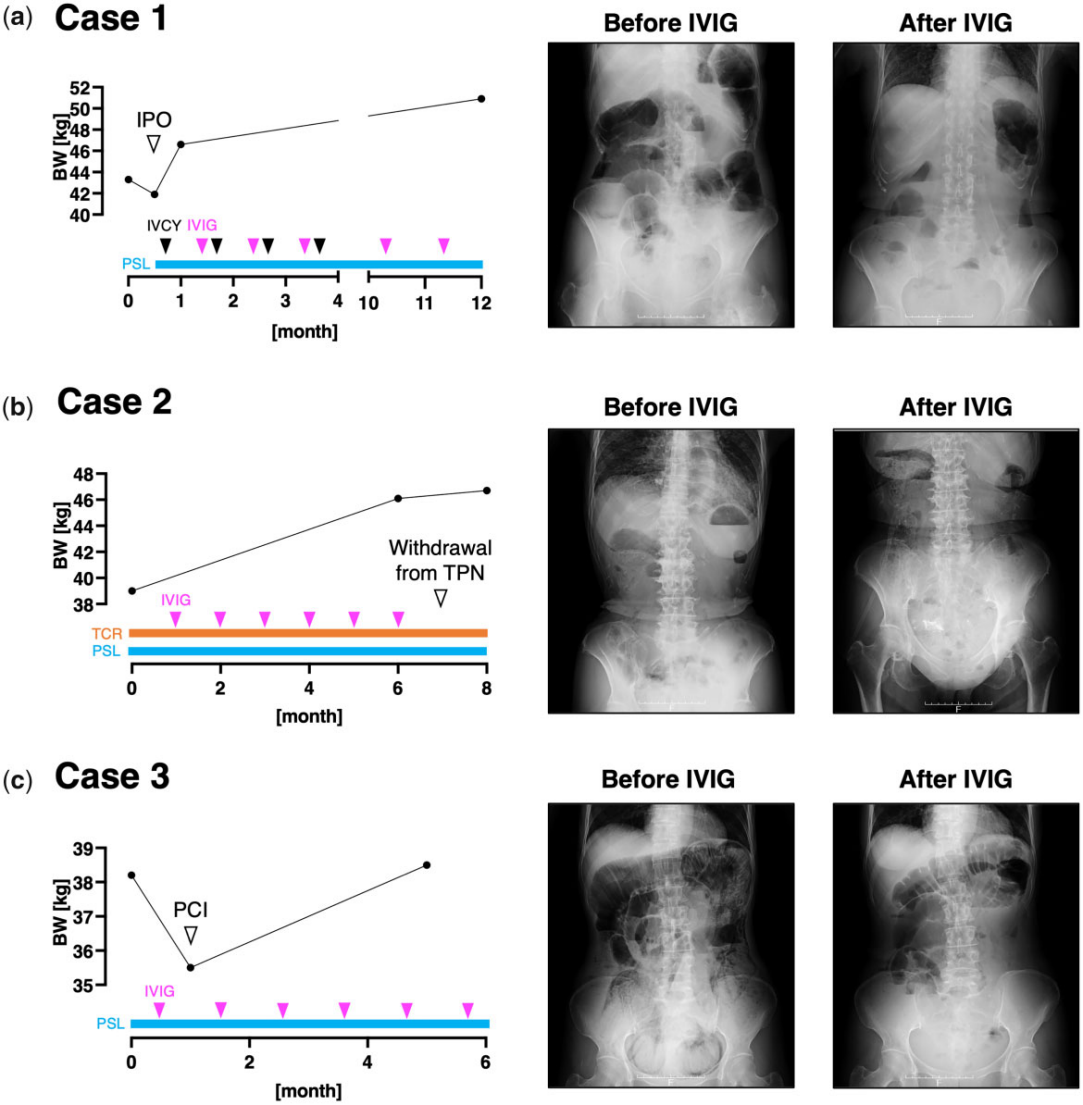
Clinical course of the three presented cases. The longitudinal change of the patients’ body weight (BW), abdominal X-ray findings at each time point of BW measurement, and the therapeutic regimen is shown. IPO: intestinal pseudo-obstruction; IVCY: intravenous cyclophosphamide; PCI: pneumatosis cystoides intestinalis; PSL: prednisolone; TCR: tacrolimus; TPN: total parenteral nutrition

A 54-year-old woman with diffuse cutaneous SSc (dcSSc) arrived at our clinic. Her disease onset was 10 years ago, accompanied by Raynaud’s phenomenon, finger stiffness and exertional dyspnoea. During the past 3 years, she had suffered from severe constipation, abdominal distention, loss of appetite and body weight loss of 20 kg. She was on esomeprazole 20 mg/day for gastroesophageal reflux and was using oral nutritional supplements because of malnutrition. Serologic investigation revealed a positive anti-nuclear antibody (Ab) at a titre of 1:2560 with a nucleolar pattern, whereas the result of disease-specific autoantibody screening was unremarkable. CT imaging of the lung showed bilateral ground-glass opacity, compatible with interstitial lung disease (ILD). MRI also demonstrated high-intensity signals around her left hip joint by short-TI inversion recovery sequence, indicating the concurrence of myositis. We intended to evaluate the GI involvement by colonoscopy, but unfortunately, pre-treatment with oral laxatives triggered IPO. We temporarily stopped her oral intake and started oral prednisolone and monthly intravenous cyclophosphamide (IVCY). However, her GI symptoms did not improve. After 1 month, we additionally introduced monthly IVIG (2 g/kg/cycle over 5 days) to manage her myositis. Surprisingly, the intestinal peristalsis increased even during the first administration of IVIG, and her intestinal gas retention immediately decreased (Fig. 1a). Over the following month, she successfully re-started oral meals and returned to her house. While she repeated IVCY for six courses and IVIG for 12 courses, her body weight increased from 41.9 kg to 50.9 kg with satisfaction of treatment outcome. Rather, she offered to extend the interval of IVIG due to the excessive frequency of defecation.

### Case 2

A 71-year-old woman with dcSSc was referred to our scleroderma centre. The disease onset was 7 years ago with Raynaud’s phenomenon and shortness of breath. She was now mainly complaining of dysphagia, anorexia, constipation and unintentional body weight loss. Those symptoms were resistant to oral prednisolone 8 mg/day, tacrolimus 2 mg/day, esomeprazole 20 mg/day, and multiple oral laxatives including magnesium oxide and sennoside. Other concomitant oral medications included tocopherol nicotinate, limaprost alfadex, teprenone and polaprezinc. Indirect immunofluorescence study demonstrated positive anti-nuclear Ab at a titre of 1:320 with homogeneous, speckled and nucleolar patterns. Serologic tests additionally revealed positivity of anti-topoisomerase I Ab and anti-SS-A Ab. She also had ILD revealed by a CT scan, and myositis in her extremities demonstrated by MRI. We introduced TPN via an implanted central venous access port to improve her malnutrition and additionally started monthly IVIG (2 g/kg/cycle over 5 days) to manage her ILD and myositis. Her dysphagia remarkably improved, and she successfully increased the amount of oral intake. Abdominal X-ray imaging also demonstrated a decrease of intestinal gas, indicating improvement of SSc-IPO (Fig. 1b). Although she had to remove her CV access port due to a catheter-related bloodstream infection after eight courses of IVIG, she could withdraw from TPN without recurrence of IPO and dysphagia.

### Case 3

A 62-year-old woman with dcSSc arrived at our scleroderma centre. She firstly noticed Raynaud’s phenomenon and puffy fingers 5 years ago, and her current chief complaint was severe abdominal distention caused by SSc-IPO. Her condition was unresponsive to several proton pump inhibitors, H2 blockers, probiotics or antibiotics rotation. Her concurrent medication included tocopherol nicotinate 300 mg/day and butyric acid bacteria. Anti-nuclear Ab showed a nucleolar pattern at a titre of 1:2560, and thorough review for disease-specific autoantibody revealed anti-U3-RNP Ab positivity. MRI of the heart revealed pericardial effusion, suggesting the existence of myocarditis. Therefore, we admitted her and started oral prednisolone and monthly IVIG (2 g/kg/cycle over 5 days), which resulted in bowel movement activation and resolution of abdominal distention. When she returned to our hospital for the second course of IVIG, an abdominal X-ray unveiled PCI (Fig. 1c). We stopped her oral ingestion and started TPN for intestinal rest. Rapid improvement in bowel motility after IVIG administration was reproducibly observed during the following courses of IVIG, but the efficacy always weaned during the drug interval. She gradually re-started oral meals and her body weight increased from 35.5 kg to 38.5 kg within half a year.

## Discussion

In this report, we described three cases of SSc-myositis overlap syndrome, in whom rapid and drastic improvement of GI symptoms, including SSc-IPO, was achieved after IVIG administration. All the patients escaped from low nutritional status represented by body weight increase and weaning from TPN. Of note, their GI symptoms were resistant to combination therapy with immunomodulators and prokinetic agents before introducing IVIG. Our case series would bring hope to SSc patients suffering from severe GI symptoms refractory to standard care.

To summarize the up-to-date insights regarding the efficacy of IVIG for GI symptoms of SSc, we conducted a systematic literature review in accordance with the PRISMA 2020 statement [6]. The process of our data collection is shown in Supplementary Fig. S1 (available at *Rheumatology* online) in a flow diagram format. We searched the PubMed database for articles using the keywords ‘IVIG’ and ‘SSc’. Only primary sources published before December 2022 were recruited into our review. Two articles were excluded because they were written in non-English languages. We screened 29 articles and found three retrospective reviews (RRs), two case series (CSs), and three case reports (CRs) describing the response of GI symptoms of SSc to IVIG administration (Table 1). All the RRs favoured the therapeutic benefit of IVIG against both upper and lower GI symptoms of SSc [4, 7, 8]. IVIG treatment was generally well tolerated, with only a few SAEs including thrombosis reported. However, these studies had several limitations. First, there would be reporting or publication biases due to their retrospective design. Second, none of the studies assigned control groups for comparing GI symptoms. Third, all the studies ended in describing long-term outcomes, failing to mention the short-term effects of IVIG highlighted in our cases. Lack of describing the short-term outcomes was also common among the CSs and CRs. Meanwhile, a CR reported a rapid improvement of GI symptoms within 2 weeks after IVIG administration, although the description was narrative and not quantitative [9].

**Table 1.** Summary of the systematic literature review

Authors, journal, published year	Study design	Country	No. of patients	IVIG dosage	Disease specific autoantibodies	Duration of IVIG treatment (Mean [s.d.])	No. of IVIG cycles (Mean [s.d.])	IVIG treatment Interval
Sanges S *et al. Autoimmun Rev.*, 2017 [4]	RR	France	46	2 g/kg over 2-4 days	ATA (*n* = 12) ACA (*n* = 9) ARA (*n* = 2) U1-RNP (*n* = 2) U3-RNP (*n* = 2) Ku (*n* = 2) PM/Scl (*n* = 1)	14.8 (19.4) months	14.5 (18.2)	1 months (*n* = 37) 3 weeks (*n* = 2) Others (*n* = 7)
Raja J *et al. Rheumatology*., 2016 [7]	RR	UK	15	2 g/kg/cycle	ARA (*n* = 4) ATA (*n* = 3) U3-RNP (*n* = 1)	29.7 (37.6) months	NA	4 weeks to 4 months
Poelman CL *et al. J Rheumatol.*, 2014 [8]	RR	USA	30	2 g/kg/cycle	ARA (*n* = 14) ATA (*n* = 5) RNP (*n* = 2)	9.4 (6.9) months	8.5 (5.3)	Typically, 1 month
Srikantharajah D *et al. Rheumatol Int.*, 2022	CS	UK	2	2 g/kg over 5 days	PM/Scl (*n* = 2)	6 weeks (*n* = 1) 5 weeks (*n* = 1)	6 (*n* = 1) 2 (*n* = 1)	NA
Clark KEN *et al. Clin Exp Rheumatol.*, 2015	CS	UK	2	2 g/kg over 5 days	ARA (*n* = 1) U3-RNP (*n* = 1)	11.5 months or 5 months, respectively.	NA	NA
Chinniah KJ and Mody GM, *Afr Health Sci.*, 2017	CR	South Africa	1	NA	NA	2 months	2	1 month
Kamei R *et al. Clin Exp Rheumatol.*, 2017 [9]	CR	Japan	1	2 g/kg over 5 days	NA	NA	1	NA
Abelha-Aleixo J *et al. Acta Rheumatol Port.*, 2015	CR	Portugal	1	2 g/kg over 5 days	NA	1 year	NA	1 month
Matsuda KM *et al.* 2023	CS	Japan	3	2 g/kg over 5 days	ATA (*n* = 1) U3-RNP (*n* = 1)	11 months (*n* = 1) 6 months (*n* = 2)	11 (*n* = 1) 6 (*n* = 2)	1 month

Concomitant immunomodulators	Relief of GI symptoms	SAEs	Other AEs
Oral corticosteroids (*n* = 39) MTX (*n* = 14) MMF (*n* = 12) AZA (*n* = 6) IVCY (*n* = 5) RTX (*n* = 2) HCQ (*n* = 2) IV corticosteroids (*n* = 1) D-penicillamine (*n* = 1)	A trend for an improvement of GERD (68% *vs* 53%, *P* = 0.06) and bowel symptom (42% *vs* 27%, *P* = 0.06) was observed from baseline to last follow-up evaluation.	Deep vein thrombosis (*n* = 1) Diffuse edematous syndrome (*n* = 1)	Fevers and chills (*n* = 3) Skin rash (*n* = 3) Headaches (*n* = 3).
PSL (*n* = 13) MMF (*n* = 9) HCQ (*n* = 4) MTX (*n* = 3)	The UCLA SCTC GIT 2.0 score significantly decreased from baseline to end of study (total: 1.07 *vs* 0.60, *P* = 0.002; reflux: 1.52 *vs* 0.88, *P* = 0.011; distention: 1.86 *vs* 1.08, *P* = 0.003, diarrhea: 0.73 *vs* 0.36, *P* = 0.026).	None reported	None reported
MMF (*n* = 21) PSL (*n* = 10) MTX (*n* = 3) CYC (*n* = 6) HCQ (*n* = 1) Imatinib (*n* = 1)	There was no statistically significant improvement in gastrointestinal severity scores defined by modified Medsger severity scales from baseline to 12 months (*P* = 0.182).	Aseptic meningitis (*n* = 1) Transient ischemic attack (*n* = 1) Acute kidney injury (*n* = 1)	Headache (*n* = 12) Fatigue (*n* = 7) Nausea/emesis (*n* = 4) Fever (*n* = 3) Arthralgias/myalgias (*n* = 3) Rash/pruritus (*n* = 3) Generalized weakness (*n* = 3) Hypertension (*n* = 2) Chest discomfort (*n* = 2) Leukopenia (*n* = 2)
mPSL (*n* = 2) PSL (*n* = 2) AZA (*n* = 2) MMF (*n* = 1)	In the first case, the patient recovered normal swallow 3 months later. In the second case, the patient regained normal weight and swallow after 3 months.	None reported	None reported
PSL (*n* = 2) CYC (*n* = 2) MMF (*n* = 2) MTX (*n* = 1)	The UCLA SCTC GIT 2.0 score improved from pre-treatment to post-treatment (2.75–2.05 and 1.69–1.25, respectively).	None reported	None reported
PSL, mPSL, CYC, AZA, RTX	The patient’s dysphagia did not respond to two courses of IVIG, and a PEG tube was inserted.	Urinary tract infection Pneumonia Sepsis around the site of the PEG tube	Diarrhea
PSL, mPSL, CyA	The patient’s gastrointestinal symptoms drastically improved for 2 weeks.	None reported	None reported
PSL, mPSL, MTX	The patient showed progressive improvement of dysphagia with a total dose of 80 g.	Aspiration pneumonia Severe respiratory failure	None reported
PSL (*n* = 3) IVCY (*n* = 1) TCR (*n* = 1)	Immediate improvement of GI symptoms and body weight increase over several months was observed in all the cases.	None reported	Frequent defecation (*n* = 1)

The contents of the previously published reports mentioning to the clinical outcome of systemic sclerosis-associated gastrointestinal (GI) symptoms against IVIG administration and the present case series are summarized.

AE: adverse event; ARA: anti-RNA polymerase III antibody; ATA: anti-topoisomerase I antibody; CR: case report; CS: case series; CyA: ciclosporin; GERD: gastro-esophageal reflux disease; IVCY: intravenous cyclophosphamide; N: number; NA: not available; PEG: percutaneous endoscopic gastrostomy; RR: retrospective review; PSL: methylprednisolone; RTX: rituximab; SAE: severe adverse event; TCR: tacrolimus; UK: United Kingdom; USA: United States of America.

Previous investigations have indicated multiple mechanisms by which IVIG exerts its beneficial properties. IVIG seems to modulate the pro-fibrotic phenotype of fibroblasts; an experimental study showed that IVIG treatment decreased the expression of fibrotic markers such as procollagen, transforming factor-beta, alpha-smooth muscle actin, and matrix metalloproteinase in SSc fibroblasts [10]. However, such cell-mediated mechanisms would take weeks or months to express the benefits. In fact, a trial demonstrated that IVIG administration improved skin scores in a short period of time [11]. An alternative idea is that IVIG might neutralize pathogenic autoantibodies in SSc. Previous reports have described the existence of autoantibodies targeting muscarinic-3-acetylcholine receptors expressed on the smooth muscle cells in the GI tract in the sera of SSc patients with GI symptoms [12, 13], which demonstrate significant inhibition of muscarinic-3-acetylcholine receptors *in vitro* [14]. Given the immediate improvement of GI symptoms after IVIG administration, such molecular-targeted mechanisms may rather play a major role in its action.

The presented cases shared known profiles of patients at high-risk of severe GI involvements including SSc-IPO, e.g. concurrence of myopathies [12, 15–17], diffuse skin sclerosis [17], nucleolar patterns of antinuclear Ab [15] and myositis-related autoantibodies such as anti-U3-RNP Ab [15]. Review of the medical records could not find any other triggers of GI dysfunction, such as relevant medications or electrolyte disturbances [18]. Among the observed risk factors, presence of myopathies is probably one of the reasons why IVIG demonstrated remarkable efficacy in our cases. This idea is supported by simultaneous improvement of GI symptoms and myopathies after IVIG administration in a previous study [7], which suggests shared pathologic mechanisms and responsiveness to IVIG between myopathies and GI symptoms in SSc-myositis overlap. It should also be noted that all the presented cases showed a nucleolar pattern of antinuclear Ab. These results can be explained by anti-topoisomerase I Ab or anti-U3-RNP Ab in Case 2 and 3, whereas no attributable autoantibodies were found in Case 1. There might be any novel autoantibodies targeting nucleolar antigens associated with severe GI manifestations or favourable response against IVIG, which would be an attractive prospect for future research.

Limitations of our present study include its retrospective design, small number of the subjects, and lack of objective and quantitative metrics for evaluating GI symptoms. In addition, concomitant use of systemic corticosteroids in all three cases made it difficult to clarify the therapeutic potential of IVIG alone. This drawback was also common among the literature listed up in our systematic review (Table 1). Furthermore, it was difficult to strictly distinguish the contribution of SSc or myositis to the GI symptoms, as was also mentioned in previous reports [4, 7].

In future studies, investigators should focus more on short-term outcomes to properly assess the efficacy of IVIG upon GI symptoms, ideally measured by reliable quantitative measures in a multicentre randomized placebo-controlled setting, recruiting a larger number of SSc patients both with and without myopathies. Novel development or translation and validation of established scoring systems, such as the UCLA SCTC GIT 2.0 score [19], should also be attempted for use in clinical trials. In addition, whether improvement of GI symptoms can be achieved by IVIG alone or only when combined with corticosteroids must be further investigated. Finally, physicians should keep in mind that the therapeutic effect of IVIG seems to be momentary. Combination with other agents of disease-modifying potential, including rituximab [20], should be considered in the long run.

## Supplementary Material

kead093_Supplementary_Data

## Data Availability

The data underlying this article cannot be shared publicly for the privacy of individuals that participated in the study. The data will be shared on reasonable request to the corresponding authors.
