# Supplementary material for: Rapid improvement of systemic sclerosis-associated intestinal pseudo-obstruction with intravenous immunoglobulin administration
Source: Rheumatology (Oxford). 2023 Feb 24;62(9):3139–45. doi: 10.1093/rheumatology/kead093 (PMC10473276; doi:10.1093/rheumatology/kead093)

**Supplementary Figure S1. Flow diagram illustrating our scheme of the systematic literature review.** SSc: systemic sclerosis, IVIG: intravenous immunoglobulin, GI: gastrointestinal, RR: retrospective review, CS: case series, CR: case report.


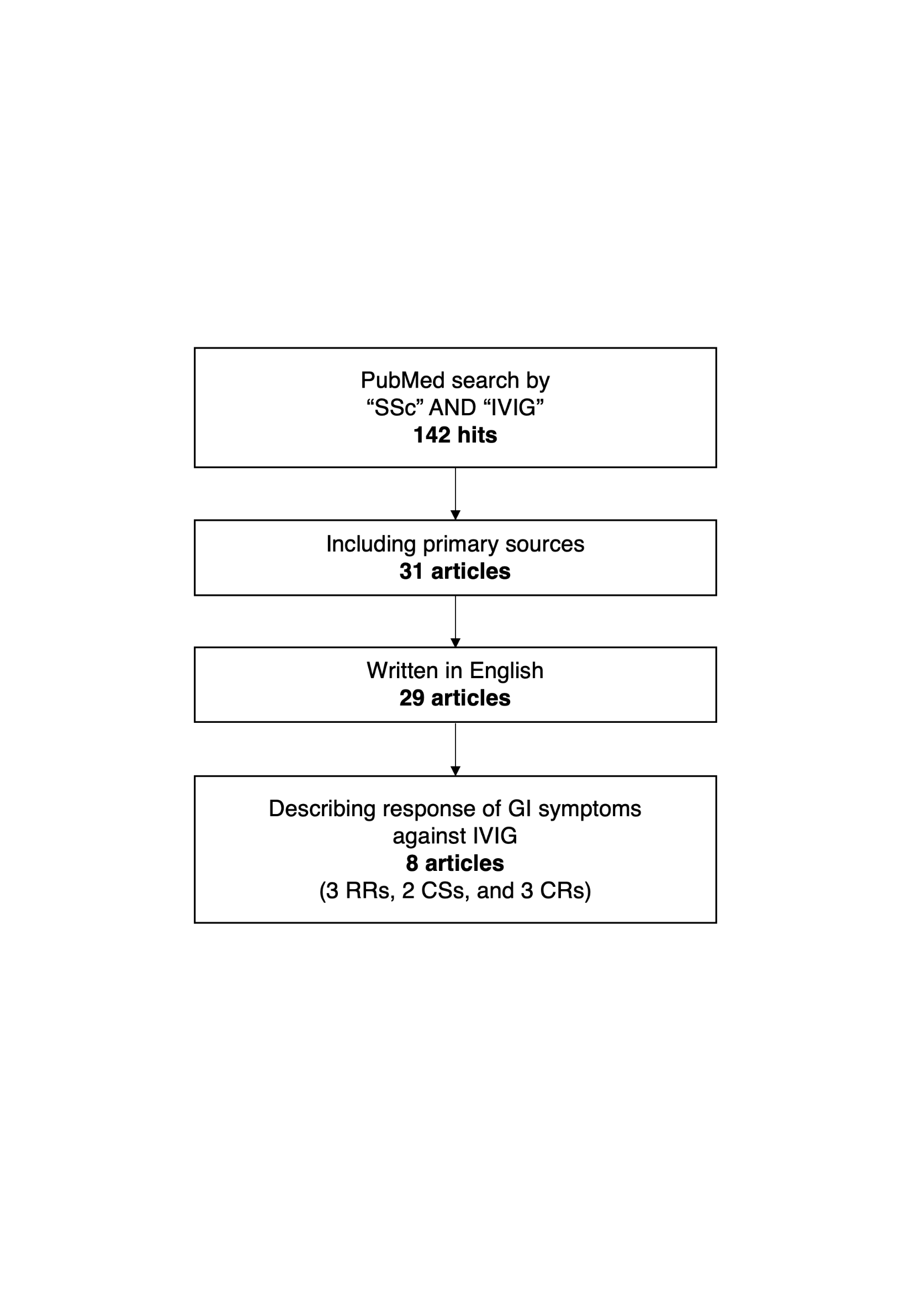

Supplement: kead093_Supplementary_Data [file kead093_supplementary_data.docx]
